# HAEMOcare: The First International Epidemiological Study Measuring Burden of Hemophilia in Developing Countries

**DOI:** 10.1055/s-0039-1688414

**Published:** 2019-06-27

**Authors:** Naresh Gupta, Abderrahmane Benbouzid, Meriem Belhani, Mohammed El Andaloussi, Khadija Maani, Yasser Wali, Soraya Benchikh El Fegoun, Hossam Ali Saad, Johnny Mahlangu

**Affiliations:** 1 Department of Medicine and The Haemophilia Centre, Maulana Azad Medical College & Associated Lok Nayak and GB Pant Hospitals, New Delhi, India; 2 Department of Orthopaedics, University Medical Centre of Ben Aknoun, Ben Aknoun, Algeria; 3 Department of Haematology, University Hospital of Beni Messous, Beni Messous, Algeria; 4 Paediatric Orthopaedic and Traumatology Service, Abderrahim Harouchi Hospital, Part of the Ibn Rochd University Hospital, Casablanca, Morocco; 5 Department of Paediatrics, Haematology and Oncology Unit, Abderrahim Harouchi University Children's Hospital–Medical School of Casablanca, Casablanca, Morocco; 6 Department of Child Health, Sultan Qaboos University, Muscat, Oman; 7 Department of Paediatrics, Alexandria University, Alexandria, Egypt; 8 Novo Nordisk Health Care AG, Zurich, Switzerland; 9 Novo Nordisk Inc., Plainsboro, New Jersey, United States; 10 Haemophilia Comprehensive Care Centre, Charlotte Maxeke Johannesburg Hospital, Department of Molecular Medicine and Haematology, National Health Laboratory Service and Faculty of the Health Sciences, University of Witwatersrand, Johannesburg, South Africa

**Keywords:** HAEMOcare, epidemiological study, hemophilia, inhibitors, orthopedic status, quality of life, developing countries

## Abstract

**Introduction** Optimizing hemophilia care remains challenging in developing countries. Burden-of-disease studies are important to develop strategies for improving hemophilia care.

**Aim** The HAEMOcare study evaluated the factors contributing to hemophilia-related orthopedic disease burden in developing countries.

**Methods** HAEMOcare was a noninterventional, cross-sectional, epidemiological study conducted in Algeria, India, Morocco, Oman, and South Africa. Male patients with severe hemophilia ( *N* = 282) aged ≥6 years, without or with inhibitors, being treated on-demand for bleeding were included. Hemophilia-related orthopedic clinical and functional status was assessed using the Hemophilia Joint Health Score (HJHS), radiological status with the Pettersson Score, and quality of life with the EuroQol five-dimension questionnaire (EQ-5D-3L). Direct and indirect economic costs of hemophilia care were also calculated.

**Results** Patients (mean [standard deviation, SD] age: 20.8 [10.6] years) experienced a mean annualized bleeding rate of 25.8. Overall mean (SD) HJHS and Pettersson score were 17.9 (12.8) and 15.0 (13.5), respectively; scores were similar between patients without or with inhibitors ( *p* = 0.21 and 0.76, respectively). Approximately 70% of adults reported problems relating to pain/discomfort and mobility parameters in the EQ-5D-3L. Mean distance to a hemophilia treatment center (HTC) was 79.4 km. As expected, total costs of hemophilia were statistically significantly higher in patients with inhibitors versus without inhibitors ( *p* = 0.002).

**Conclusion** Inadequate access to HTCs and expert care, along with high bleeding rates, led to equivalent hemophilia-related orthopedic morbidity between hemophilia patients without and with inhibitors. HAEMOcare documented the economic and disease burdens associated with suboptimal hemophilia care in developing countries.

## Introduction

Modern management has significantly improved the clinical course of hemophilia. However, the development of inhibitors against clotting factors and chronic arthropathy remains major causes of morbidity. 1 In developing countries, with limited resources and limited or no access to any treatment, optimizing hemophilia care services can be beneficial for all patients.

In developed countries, large-scale observational studies have evaluated hemophilia-related orthopedic status and outcomes such as quality of life (QoL) and resource consumption, to consider risk factors for poor outcomes and improve disease management. 2 3 4 5 6 7 Few studies have been reported from developing countries. 8 9 10 Burden-of-disease studies are needed in developing nations to prioritize determinants of hemophilia care and to formulate adapted management strategies to improve outcomes.

The HAEMOcare study was conducted in five developing countries (defined by World Bank Atlas Method as low–middle income 11 ) to identify the unmet needs of severe hemophilia patients, including exploring the possible relation of hemophilia-related orthopedic status to inhibitors, treatment history, hemophilia management, assessment of QoL, and evaluation of the economic aspects of hemophilia. 12

## Methods

### Study Design

HAEMOcare (NCT01503567) was a multicenter, noninterventional, cross-sectional, epidemiological study in Algeria, India, Morocco, Oman, and South Africa, with a design similar to a European study. 2 HAEMOcare was conducted according to the Declaration of Helsinki, Good Clinical Practice as set out by the International Conference on Harmonisation of Technical Requirements for Registration of Pharmaceuticals for Human Use, and applicable national guidelines, with local institutional review board/independent ethics committee approval obtained accordingly. Before enrolment, written informed consent was obtained from each patient, or a legally acceptable representative. Recruitment and study-related assessments were conducted over an 8-month period. Every study participant had one visit which included all study-related assessments.

### Study Population

Eligible patients were enrolled between January 2, 2012 and September 3, 2012. Included patients were males aged ≥6 years, with severe congenital hemophilia A or B (FVIII or FIX levels <1 international unit [IU]/dL or <1% of normal), without or with inhibitors and receiving hemostatic treatment on-demand. Those with other known clinically relevant coagulation disorders, receiving prophylactic hemophilia treatment, or receiving treatment for hepatitis C or human immunodeficiency virus infection were excluded.

The enrolment target was 300 patients, with 50 to 70 from each participating country. Subgroup analyses were stratified by age and inhibitor status: pediatric patients (6–18 years) without inhibitors; pediatric patients with inhibitors; adult patients (>18 years) without inhibitors; and adult patients with inhibitors.

### Objectives

The primary objective of HAEMOcare was to evaluate the orthopedic status and degree of arthropathy in severe hemophilia A and B patients without or with inhibitors in developing countries. Hemophilia-related orthopedic status was assessed clinically, using the Hemophilia Joint Health Score (HJHS) administered by a physical therapist, and radiologically, using the Pettersson Score; higher scores represented a worse status in both scales.

Secondary objectives were to evaluate the relationship of previous disease management to current disease status, patient QoL, and the economic burden associated with hemophilia treatment. The generic EuroQol five-dimension questionnaire (three-level version; EQ-5D-3L), including the 100-point visual analogue scale (VAS), was used to assess QoL. 13 Economic burden was determined for the 12 months before the study visit by measuring direct expenses (treatment and transportation costs), indirect expenses (lost patient/family productivity), and capacity to cover expenses (insurance status; socioeconomic status of the patient/family) using a predesigned, structured questionnaire. The primary investigator at each site determined occupational and educational status locally, per country standards. Home treatment (full or partial) was at the discretion of the treating physician. Target joints were commonly identified per the International Society on Thrombosis and Hemostasis (ISTH) definition as joints in which three or more spontaneous bleeds had occurred within a 6-month period. 14

### Statistical Analyses

The prespecified enrolment targets would allow detection of an effect size of 0.40 in the Pettersson score, with a two-sided α of 0.05 and 90% power, and an effect size of 0.5 with a power >80% when controlling for ≤8 covariates in a multiple regression model. Missing data were not imputed; however, when X-rays were unavailable for assessment of Pettersson score, results were imputed as 0. Results were comparable between observed and imputed Pettersson scores for all analyses, therefore data for observed Pettersson score are reported here.

The association of inhibitor status with Pettersson score, HJHS, and EQ-5D-3L VAS was analyzed using a multivariate linear regression model, adjusting for age, insurance status, access to expert orthopedic consultation, regular sports participation, and regular physical therapy sessions. Direct and indirect economic burdens were assessed with a general linear model adjusting for age, inhibitor status, Pettersson score, and HJHS. Descriptive values are reported as mean with standard deviation (SD). Values from statistical analysis are reported as adjusted estimated difference (AED) and 95% confidence interval (CI) for Pettersson score and HJHS, while EQ-5D-3L VAS scores are reported as adjusted mean difference (AMD) and 95% CI. SAS 9.1 software (SAS Institute, Cary, NC, United States) was used for all analyses.

## Results

### Study Population

Patient demographics are shown in Table 1 . Of 282 patients enrolled (250 [88.6%] hemophilia A, 32 [11.4%] hemophilia B), 50 (17.7%) patients had inhibitors. The pediatric group (6–18 years) included 128 patients, 104 (81.2%) patients without and 24 (18.8%) with inhibitors; the adult group included 154 patients, 128 (83.1%) patients without and 26 (16.9%) with inhibitors. Family history of inhibitors was more common in patients with inhibitors than those without. Hemophilia was diagnosed at a mean age of 34 months, and the presence of inhibitors at a mean age of 175 months (14.6 years).

**Table 1:** Demographic characteristics of the HAEMOcare study population

	Pediatric (6–18 y)		Adult (>18 y)		
	Without inhibitors ( *n =* 104)	With inhibitors ( *n =* 24)	Without inhibitors ( *n =* 128)	With inhibitors ( *n =* 26)	Total ( *N =* 282)
Country of enrolment, *n* (%)					
Algeria	11 (11)	7 (29)	32 (25)	10 (38)	60 (21)
India	31 (30)	4 (17)	40 (31)	5 (19)	80 (28)
Morocco	43 (41)	7 (29)	10 (8)	0	60 (21)
Oman	19 (18)	6 (25)	26 (20)	2 (8)	53 (19)
South Africa	0	0	20 (16)	9 (35)	29 (10)
Mean age at enrolment, years (SD)	11.8 (3.6)	12.3 (3.8)	28.5 (9.2)	27.0 (5.8)	20.8 (10.6)
Race, *n* (%)					
White	54 (52)	14 (58)	48 (38)	12 (46)	128 (45)
Black/African	0	0	14 (11)	6 (23)	20 (7)
Asian	31 (30)	4 (17)	41 (32)	6 (23)	82 (29)
Other	19 (18)	6 (25)	25 (20)	2 (8)	52 (18)
Marital status, *n* (%)					
Married	0	0	27 (29)	3 (12)	40 (14)
Unmarried	104 (100)	24 (100)	91 (71)	23 (88)	242 (86)
Occupational and educational status, a *n* (%)					
Above local average	33 (32)	8 (33)	33 (26)	7 (27)	81 (29)
Local average	51 (49)	13 (54)	49 (38)	7 (27)	120 (43)
Below local average	20 (19)	3 (13)	46 (36)	12 (46)	81 (29)
Family history of hemophilia, *n* (%)					
Yes	66 (63)	17 (71)	91 (71)	19 (73)	193 (68)
No	38 (37)	7 (29)	37 (29)	7 (27)	89 (32)
Family history of inhibitors, *n* (%)	( *n =* 66)	( *n =* 17)	( *n =* 91)	( *n =* 19)	( *n =* 193)
Yes	6 (9)	5 (29)	3 (3)	6 (32)	20 (10)
No	58 (88)	10 (59)	86 (95)	12 (63)	166 (86)
Unknown	2 (3)	2 (12)	2 (2)	1 (5)	7 (4)
Type of hemophilia, *n* (%)					
A	96 (92)	24 (100)	104 (81)	26 (100)	250 (89)
B	8 (8)	0	24 (19)	0	32 (11)
Mean time since diagnosis, months (SD)	( *n =* 100) 121.3 (43.1)	( *n =* 23) 138.2 (44.2)	( *n =* 123) 300.2 (122.1)	( *n =* 22) 287.6 (97.8)	( *n =* 268) 218.5 (126.4)
Mean time since diagnosis of inhibitors, months (SD)	–	( *n =* 24) 36.6 (27.5)	–	( *n =* 25) 85.8 (68.4)	( *n =* 49) 61.7 (57.6)
Average bleeds per month during prior 12 months, mean (SD)	( *n =* 86) 2.0 (1.8)	( *n =* 22) 2.0 (1.7)	( *n =* 124) 2.3 (2.0)	( *n =* 26) 2.0 (1.5)	( *n =* 258) 2.2 (1.9)
Presence of target joints, *n* (%)	89 (86)	18 (75)	112 (88)	17 (65)	236 (84)
Insurance/incapacity benefits, *n* (%)					
Fully reimbursed	37 (36)	15 (63)	71 (55)	16 (62)	139 (49)
Partially reimbursed	10 (10)	3 (13)	7 (5)	2 (8)	22 (8)
Not reimbursed	57 (55)	6 (25)	50 (39)	8 (31)	121 (43)

a The primary investigator, according to country standards, determined average occupational and educational status locally.

### Orthopedic Assessments

**Fig. 1:**
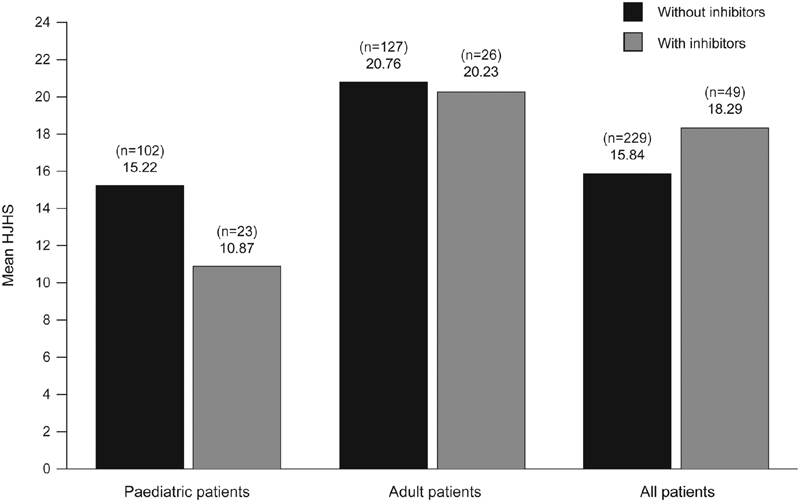
Mean total Hemophilia Joint Health Score in patients without and with inhibitors.

**Fig. 2:**
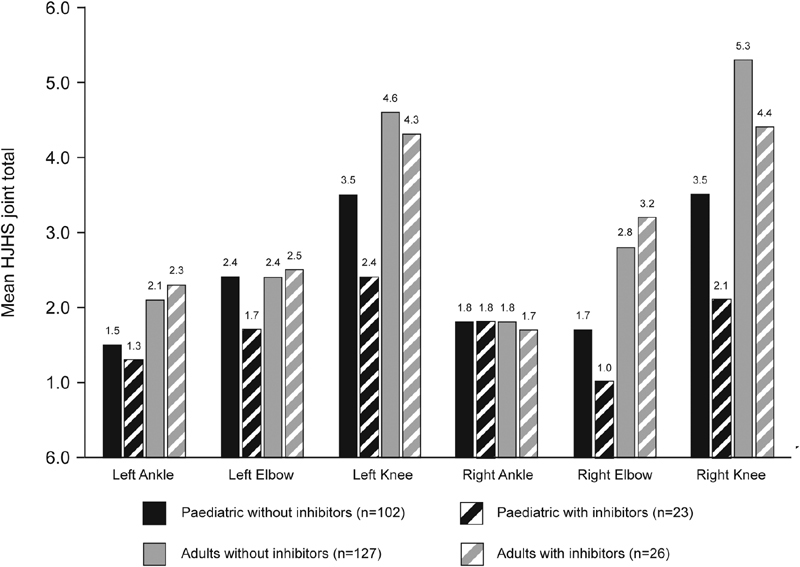
Mean HJHS joint scores in patients without and with inhibitors. HJHS, Hemophilia Joint Health Score.

**Fig. 3:**
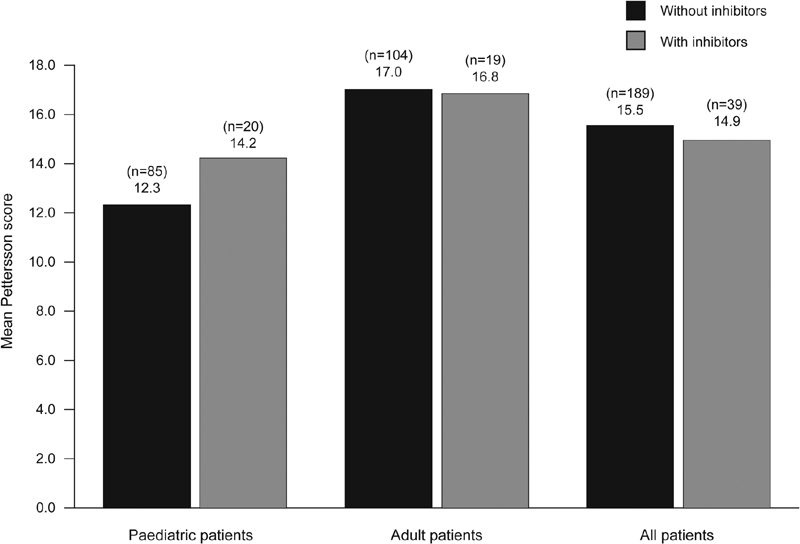
Mean observed Pettersson score among patients without and with inhibitors.

**Fig. 4:**
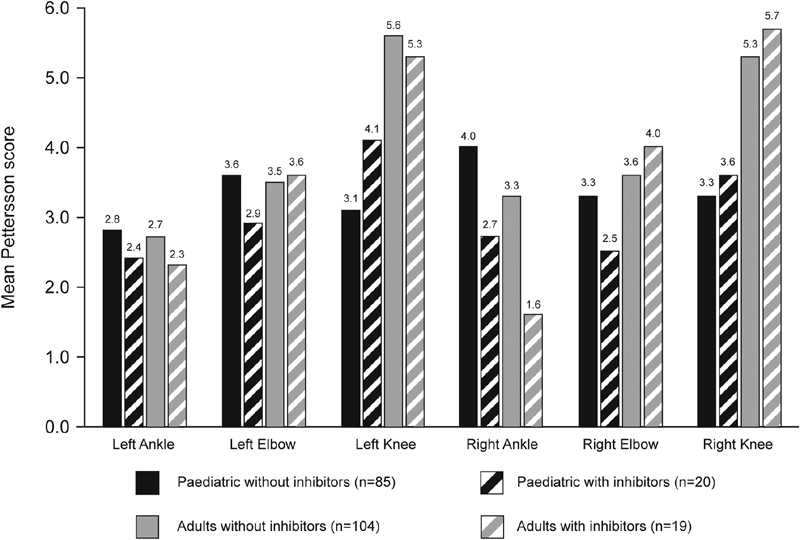
Mean observed Pettersson joint scores in patients without and with inhibitors.

Mean (SD) total HJHS, HJHS global gait scores, and observed Pettersson scores are shown in Table 2 . Hemophilia-related orthopedic disabilities limited the HJHS in 70% of patients. Mean overall AED in HJHS was similar between patients without and with inhibitors (AED: –2.45 [95% CI: –6.30, 1.40]; *p* = 0.21; Fig. 1 ), with no significant differences in bilateral HJHS at the ankle, elbow, or knee ( Fig. 2 ). Overall mean (SD) HJHS global gait score was also similar between patients without and with inhibitors (1.54 [1.32] and 1.35 [1.38], respectively). In addition, overall mean observed Pettersson score was similar between patients without and with inhibitors (AED: 0.72 [95% CI: –3.91, 5.35]; *p* = 0.76), and across subgroups ( Fig. 3 ). There were no significant differences in observed Pettersson scores between any of the patient subgroups ( Fig. 4 ).

**Table 2:** Summary of mean total Pettersson score, HJHS, and global gait assessment (HJHS) a

	Pediatric (6–18 y)		Adult (>18 y)		
	Without inhibitors ( *n =* 104)	With inhibitors ( *n =* 24)	Without inhibitors ( *n =* 128)	With inhibitors ( *n =* 26)	Total ( *N* = 282)
Observed Pettersson score, mean (SD)	( *n* = 85) 12.3 (13.3)	( *n* = 20) 14.2 (19.6)	( *n* = 104) 17.0 (12.6)	( *n* = 19) 16.8 (9.7)	( *n* = 228) 15.0 (13.5)
Total HJHS, mean (SD)	( *n* = 102) 15.2 (12.2)	( *n* = 23) 10.9 (13.0)	( *n* = 127) 20.8 (13.1)	( *n* = 26) 20.2 (9.1)	( *n* = 278) 17.9 (12.8)
Skills not within normal limits, *n* (%)					
0	47 (45.2)	15 (62.5)	20 (15.6)	3 (11.5)	85 (30.1)
1	20 (19.2)	4 (16.7)	28 (21.9)	7 (26.9)	59 (20.9)
2	22 (21.2)	1 (4.2)	37 (28.9)	7 (26.9)	67 (23.8)
3	7 (6.7)	1 (4.2)	21 (16.4)	5 (19.2)	34 (12.1)
4	5 (4.8)	1 (4.2)	20 (15.6)	4 (15.4)	30 (10.6)
Not applicable/missing	3 (2.9)	2 (8.3)	2 (1.6)	0	7 (2.5)

Abbreviations: HJHS, Hemophilia Joint Health Score; SD, standard deviation.

a Pettersson score, HJHS total, and HJHS global gait score were not reported by physicians for all patients.

Mean (SD) HJHS was significantly lower in pediatric patients (10.19 [1.57]) than in adults (17.25 [1.58]; AED: 7.05 [95% CI: 4.08, 10.00]; *p* < 0.001). Mean (SD) observed Pettersson score was also significantly lower in pediatric patients (9.41 [1.80]) than in adults (15.41 [1.81]; AED: 6.00 [95% CI: 2.47, 9.53]; *p* < 0.001).

Of 281 patients assessed, 236 (84%) patients had target joints; the most common target joints were the knees (right, 53.4%; left, 52.2%). Mean annualized bleeding rate (ABR) was 25.8 and similar between patients without or with inhibitors ( *p* > 0.05).

### Management of Hemophilia

In the 12 months prior to assessment, 206/232 patients without inhibitors (88%) received on-demand treatment with FVIII or FIX. Of 50 patients with inhibitors, 21 patients (42%) reported use of activated recombinant factor VII (rFVIIa), 10 patients (19%, all adults) reported use of activated prothrombin complex concentrate (aPCC), and 19 patients (38%) reported use of FVIII concentrates, including ongoing immune tolerance induction (ITI) in one pediatric patient. Some patients reported the use of multiple agents and one pediatric patient without inhibitors received a single dose of rFVIIa 90 µg/kg ( Table 3 ).

**Table 3:** Summary of antihemophilia therapy doses administered to patients in the 12 months prior to enrolment a

**Patients without inhibitors**	**Pediatric (** ***n*** ** = 104)**	**Adult (** ***n*** ** = 128)**	**Total (** ***n*** ** = 232)**
FVIII (IU/kg)			
* n* (%)	81 (78)	100 (78)	181 (79)
Mean (SD)	4,172.26 (9,462.27)	20,698.59 (67,070.87)	13,302.83 (50,812.12)
FIX (IU/kg)			
* n* (%)	4 (4)	21 (16)	25 (11)
Mean (SD)	3,469.50 (3,540.1)	15,260.19 (28,541.23)	13,373.68 (26,454.94)
**Patients with inhibitors**	**Pediatric (** ***n*** ** = 24)**	**Adult (** ***n*** ** = 26)**	**Total (** ***n*** ** = 50)**
rFVIIa (µg/kg)			
* n* (%)	9 (38)	12 (46)	21 (42)
Mean (SD)	2,316.22 (2,548.46)	16,141.00 (34,074.10)	10,216.10 (26,273.91)
aPCC (IU/kg)			
* n* (%)	–	10 (38)	10 (19)
Mean (SD)	–	28,536 (34,618.22)	28,536.00 (34,618.22)
FVIII (IU/kg)			
* n* (%)	13 (54)	6 (23)	19 (38)
Mean (SD)	1,614.77 (1,449.57)	1,156.17 (1520)	1,469.95 (1,445.89)

Abbreviations: aPCC, activated prothrombin complex concentrate; IU, international unit; rFVIIa, activated recombinant factor VII; SD, standard deviation.

Note: one pediatric patient without inhibitors received a single dose rFVIIa 90 µg/kg and nine patients without and one patient with inhibitors received “other” hemophilia treatment (specific details of treatment not recorded).

a Numbers reported in the table do not match the total values for each group due to missing data and some patients receiving multiple therapies.

Mean (SD) doses of hemostatic treatment during the 12 months prior to enrolment are reported in Table 3 . Among adult patients with inhibitors, patients were treated mainly with bypassing agents rFVIIa (46%) and aPCC (38%). In pediatric patients with inhibitors, rFVIIa was the only bypassing agent used (nine patients [38%]).

Of 280 patients with a medical history available, 26 (9.3%) had any history of prophylaxis prior to enrolment. Prophylaxis was received by 4/104 (3.8%) pediatric patients without inhibitors, 1/24 (4.2%) pediatric patients with inhibitors, 1/26 (3.9%) adults with inhibitors, and 20/126 (15.9%) adults without inhibitors. History of previous prophylaxis was similar between patients without inhibitors (24/230 [10.4%]) and those with inhibitors prior to inhibitor development (2/50 [4.0%]; *p* = 0.19). No patients with inhibitors had received any prophylaxis with bypassing agents.

There was no difference in the proportion of hemophilia-related orthopedic surgical procedures ( *p* = 0.97), use of orthopedic aids ( *p* = 0.44), regular physical therapy sessions ( *p* = 0.86), or regular participation in sport ( *p* = 0.28) between patients without and with inhibitors. More patients with inhibitors (35; 71.4%) received prompt hemostatic treatment within the first 2 hours of bleeding compared with those without (123; 53.3%; *p* = 0.03; Table 4 ).

**Table 4:** Management of hemophilia in patients without and with inhibitors

	Without inhibitors ( *n =* 232)	With inhibitors ( *n =* 50)	Total ( *N =* 282)	*p* -Value
Practice home treatment, *n* (%)	80 (34.5)	25 (50)	105 (37.2)	0.04
Hemostatic treatment initiated <2 h after bleed onset, *n* (%)	123 (53.3)	35 (71.4)	158 (56.0)	0.03
Orthopedic surgical procedures (any type), *n* (%)	33 (14.2)	7 (14.0)	40 (14.2)	0.97
Use of orthopedic aid (any type), *n* (%) a	102 (44.0)	25 (50.0)	127 (45.0)	0.44
Regular physical therapy sessions, *n* (%)	99 (42.7)	22 (44.0)	121 (42.9)	0.86
Regular participation in sport, *n* (%)	51 (22.0)	6 (12.0)	57 (20.2)	0.28

a During the 12 months prior to assessment.

Thirty-seven percent of patients received home treatment; a greater proportion (25/50; 50%) of patients with inhibitors practiced home treatment than those without (80/232; 35%; *p* = 0.04; Table 4 ). Patients travelled a mean (SD) of 79.4 (124.0) km to reach their hemophilia treatment center (HTC), incurring a mean (SD) transportation cost of US$13.02 (47.9) per visit. Regular physician follow-up was feasible for 234/282 (83.0%) patients. Mean (SD) number of visits to the nearest HTC during the preceding 12 months was 12.8 (14.8), against an ABR of 25.8. An orthopedic expert was available to 133/282 (47.2%) patients on a regular basis and to 90 (31.9%) patients irregularly ( Table 5 ).

**Table 5:** Summary of HJHS and Pettersson scores by hemophilia care situation a

	Pettersson score (observed)	HJHS
Home treatment, mean (SD)	( *n =* 228)	( *n =* 278)
Yes ( *n =* 105)	18.49 (14.86)	19.75 (13.62)
No ( *n =* 177)	13.37 (12.50)	16.74 (12.21)
Regular physical therapy, mean (SD)	( *n =* 228)	( *n =* 278)
Yes ( *n* = 121)	14.29 (13.41)	17.90 (12.01)
No ( *n* = 161)	15.64 (13.56)	17.82 (13.43)
Regular sport, mean (SD)	( *n =* 228)	( *n =* 278)
Yes ( *n* = 57)	10.83 (10.49)	14.88 (11.07)
No ( *n* = 224)	16.06 (13.97)	18.69 (13.13)
Nonparticipant ( *n =* 1)	–	5.00
Access to an orthopedic expert, mean (SD)	( *n =* 227)	( *n =* 275)
Regular ( *n =* 133)	17.79 (15.66)	19.73 (13.74)
Irregular ( *n =* 90)	12.91 (9.90)	17.81 (11.41)
No ( *n =* 56)	12.00 (11.94)	13.83 (11.69)

Abbreviations: HJHS, Hemophilia Joint Health Score; SD, standard deviation.

a Pettersson score, HJHS and access to an orthopaedic expert were not reported by physicians for all patients.

### Relationships between Hemophilia-Related Orthopedic Status and Hemophilia Management

Mean observed Pettersson score and HJHS were similar for patients with or without: access to home care, access to expert orthopedic care, regular participation in sports, receipt of regular physical therapy ( Table 5 ).

### Quality of Life

**Fig. 5:**
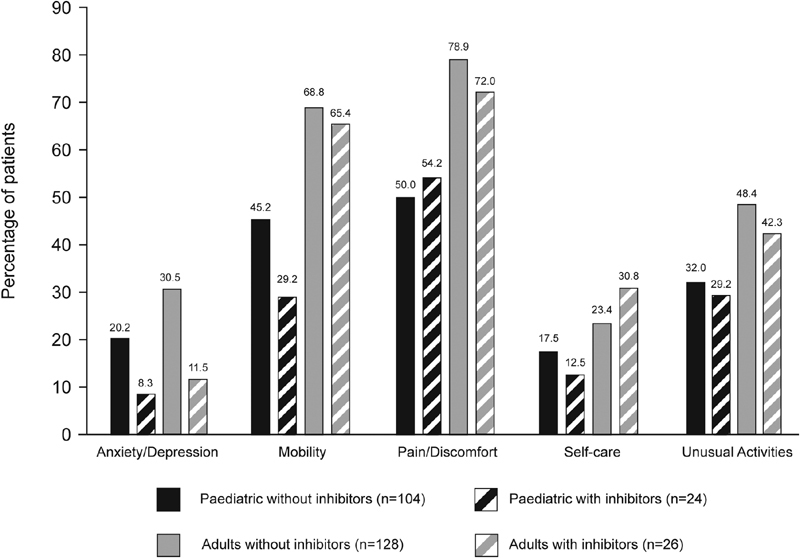
Percentage of hemophilia patients who reported having problems according to the dimensions of the EQ-5D-3L questionnaire.

Most patients reported problems on the EQ-5D-3L domains, most often in the pain/discomfort (any problems, 65.2%) and mobility (any problems, 56.4%) dimensions. Reported problems were similar between pediatric and adult patients ( Fig. 5 ). Mean EQ-5D-3L VAS scores were similar between patients without inhibitors (68.74) and with inhibitors (73.54; AMD: 3.78 [95% CI: –3.00, 10.56]; *p* = 0.27). Mean (SD) VAS scores were 67.37 (19.45) in adults without inhibitors, 70.65 (19.70) in adults with inhibitors, 70.43 (24.62) in pediatric patients without inhibitors, and 76.67 (24.12) in pediatric patients with inhibitors.

### Economic Aspects of Hemophilia

Socioeconomic data are shown in Table 1 . Occupational and educational status was average or higher for 71% of patients. Overall, mean (SD) family income/month was US$907.60 (1,246.01; median, US$350 [range, 15–7,772]) and treatment costs were fully reimbursed in 139/282 patients (49.3%), and partially reimbursed in 22/282 patients (7.8%). In Algeria, all patients, regardless of age or inhibitor status, had treatment costs fully reimbursed. In Oman, all pediatric patients, without or with inhibitors, and adult patients with inhibitors had treatment costs fully reimbursed. No reimbursement was available for 121 patients (42.9%); the majority of these 121 patients were from India (64 patients) and Morocco (41 patients).

Mean monthly direct ( Table 6 ) and indirect ( Table 7 ) costs relating to hemophilia treatment during the 12 months preceding enrolment were used to compute the combined annualized cost of managing hemophilia. The total combined mean (SD) annualized cost of hemophilia was US$19,513 (38,739) per year, ranging from $9,135 (12,196) for pediatric patients without inhibitors to $47,225 (91,203) for adults with inhibitors. Costs were significantly lower for patients without inhibitors than those with inhibitors for direct (AED: US$18,154.50 [95% CI: 6,371.71–29,937.29]; *p* = 0.003), indirect (AED: US$999.68 [95% CI: 277.76–1,721.59]; *p* = 0.007), and total costs (AED: US$18,551.43 [95% CI: 6,791.76–30,311.10; *p* = 0.002).

**Table 6:** Direct monthly costs (in US$) associated with hemostatic treatment in patients without and with inhibitors ( *n* = 255) a

	Without inhibitors	With inhibitors	Total
FVIII			
* n*	177	19	196
Mean (SD)	1,566.36 (2,389.25)	1,230.79 (1,634.91)	1,533.83 (2,325.71)
FIX			
* n*	24	–	24
Mean (SD)	952.67 (1151.32)	–	952.67 (1151.32)
rFVIIa			
* n*	1	16	17
Mean (SD)	71.0	6,424.88 (8,977.63)	6,051.12 (8,828.09)
aPCC			
* n*	–	7	7
Mean (SD)	–	2,141.00 (2,203.45)	2,141.00 (2,203.45)
Other			
* n*	9	2	11
Mean (SD)	25.44 (29.05)	20.00 (15.56)	24.45 (26.53)

Abbreviations: aPCC, activated prothrombin complex concentrate; rFVIIa, activated recombinant factor VII; SD, standard deviation.

a Direct monthly costs were not reported by physicians for all patients.

**Table 7:** Indirect consumption of patient/family and community resources during the 12 months before enrolment

	Without inhibitors	With inhibitors
Days of school absenteeism (patient)		
* N*	153	33
Mean (SD)	29.7 (36.7)	32.0 (36.1)
Working days lost (patient)		
* n*	42	8
Mean (SD)	30.8 (38.2)	69.5 (61.9)
Indirect cost of lost working days (US$, patient)		
* n*	38	7
Mean (SD)	440.9 (781.9)	3,192.6 (4,220.9)
Working days lost (family)		
* n*	59	12
Mean (SD)	19.7 (15.2)	25.8 (40.1)
Indirect cost of lost working days (US$)		
* n*	58	11
Mean (SD)	344.0 (810.9)	415.6 (541.3)

Abbreviation: SD, standard deviation.

## Discussion

The current study assessed hemophilia-related orthopedic status, QoL, and economic burden of patients currently treated on-demand in five countries. HJHS and Pettersson scores were similar between hemophilia patients without and with inhibitors, and both scores were significantly lower in pediatric patients compared with adults. In developed countries, studies have reported better hemophilia-related orthopedic status in patients without inhibitors, including the European study that inspired HAEMOcare. 2 15 Numerous studies have shown the effect of prophylaxis in improving ABR, joint symptoms, and QoL in patients without inhibitors. 15 16 17 18 The dismal results in HAEMOcare may reflect suboptimal hemophilia care for those without inhibitors in developing countries.

Notably, our study did not collect information on treatment protocols, nor was adherence to a specific/standard protocol required; patients were treated using local protocols, and patients on prophylaxis at the time of study recruitment were not included. Only 11% of adults and 4% of pediatric hemophilia patients being treated on-demand had any history of prior prophylactic therapy, considerably lower than rates of prophylaxis reported in developed countries (adults, 38–55%; pediatric patients, 41–84%). 19 20 For patients with inhibitors in our study, only two patients being treated on-demand had ever received prophylaxis and only one patient (pediatric) had received ITI.

In our study, 70% of patients being treated on-demand reported limitations on the HJHS from hemophilia-related orthopedic morbidities, consistent with previous reports. 21 Despite the overall young age (mean: 20.8 years) of patients, 84% had target joints. Patients experienced a mean ABR of 25.8, with no significant differences between adults and pediatric patients, or between those without and with inhibitors. This finding contrasts with developed countries, where substantially lower bleeding rates have been observed; in a German study, only 17% of patients receiving on-demand therapy reported more than 12 bleeds per year and 39% of patients had fewer than one bleed per year. 22 However, patients without inhibitors receiving on-demand treatment in developed countries could be expected to have only a mild bleeding phenotype, as severely bleeding patients will generally receive prophylaxis. 23 Therefore, any comparison of the patients in the current study with populations receiving on-demand treatment in developed countries requires caution. Despite this caveat, our results suggest suboptimal hemostatic treatment and orthopedic care in developing countries for patients with hemophilia. Barriers to early hemostatic treatment of bleeding events can impact hemophilia-related orthopedic disease burden; such barriers include substantial treatment costs, parent/caregiver inconvenience, distance to the HTC, and more rarely, problems related to venous access or home infusion. 24

The results of the study showed that there is a need for wider and earlier comprehensive care, despite patients in HAEMOcare receiving the best standard of care available to them at the time, as expert orthopedic care was offered mainly to patients with more advanced morbidities. Patients with regular access to orthopedic care showed numerically higher HJHS and Pettersson score than patients without, indicating that access to care may be focused on patients with more severe morbidities or that referrals to an orthopedist are late ( Table 5 ).

Similar QoL scores were found between patients with and without inhibitors. The similarity in QoL among patients, regardless of inhibitor status, may suggest that the level of hemophilia care for patients without inhibitors currently (and in most cases lifelong) treated on-demand was inadequate, and therefore their QoL was similar to that of patients with inhibitors. This similarity contrasts with results previously reported in Europe, in which the absence of inhibitors had a positive impact on QoL. 2 25

Despite similar QoL, the direct, indirect, and total costs associated with hemophilia and its treatment were, as expected, lower in patients without inhibitors than in those with inhibitors and only 57% of patients had access to full or partial medical insurance. However, FVIII and FIX concentrates and bypassing agents were not always available or accessible, which may have impacted upon the treatments received and their costs. It is important to note that these costs are purely indicative and were incurred in 2012 and there may be variations between the systems of care in each country that affect the availability and pricing of treatments. While access to hemophilia care follow-up was considered to be feasible in 83% of patients, the mean travel distance to their HTC was 79.4 km and mean transportation costs were evaluated to be around $US13 per visit (1.4% of the mean family monthly income of $US 907.60). Wider universal health coverage or individual health insurance coverage would greatly help to reduce the economic burden for hemophilia patients. The availability of treatment close to the patient or at home and referral networks to reduce travel distances would also provide economic and medical benefits to patients.

Home treatment was often initiated late in the disease course, which could explain observations of numerically higher HJHS and Pettersson score for patients who received home treatment ( Table 5 ). Patients would benefit from wider application and earlier initiation of supervised home treatment programs, as recommended in the World Federation of Hemophilia guidelines. 26

### Limitations

Patients receiving prophylaxis were excluded from HAEMOcare. It is estimated that only 1 to 31% of all patients in the study countries would have received prophylaxis at the time of the study, with the exception of Algeria. 27 Although prophylaxis estimates for Algeria had reached 90% for pediatric and 40% for adult patients in 2015, 28 it was expected that they would have been similar to other developing countries at the time of the study. Therefore, given the low rate of previous exposure to prophylaxis in patients in this study and the exclusion of patients currently receiving prophylaxis, we believe that the study population here was reflective of the rate of prophylaxis in the population of patients with severe hemophilia at the time of the study, in at least four of the five developing countries studied.

This study was only able to enroll 50 patients with inhibitors against a target of 75, limiting its statistical power. Furthermore, much of the data were collected retrospectively, and radiological scores were based on preexisting X-rays.

## Conclusions

The HAEMOcare study showed that, in the participating developing countries, inadequate access to HTCs and expert care along with high bleeding rates led to equivalent hemophilia-related orthopedic status, arthropathy, and QoL between patients without and with inhibitors. The economic burden from disease was high in terms of direct and indirect costs, and the large travel distances to access hemophilia treatment and expert consultations added to the burden of hemophilia patients. HAEMOcare documented the economic and disease burdens associated with suboptimal hemophilia care in developing countries.
